# Supplementary material for: ProteinShader: illustrative rendering of macromolecules
Source: BMC Struct Biol. 2009 Mar 30;9:19. doi: 10.1186/1472-6807-9-19 (PMC2672931; doi:10.1186/1472-6807-9-19)
Supplement: Additional file 1 — ProteinShader program without source code. This compressed file contains the complete ProteinShader program including associated libraries, but no source code. A README.txt file gives an overview of the ProteinShader distribution, and the index.html file in the help subdirectory has directions on getting started with the program as well as a set of tutorials. [file 1472-6807-9-19-S1.zip › ProteinShader-beta-0_9_4-binary/help/api/org/proteinshader/structure/Category.html]

Category (ProteinShader API)


|  |  |  |
| --- | --- | --- |
| **Overview** \| **Package** \| **Class** \| **Use** \| **Tree** \| **Deprecated** \| **Index** \| **Help** | |  |
| **PREV CLASS**   **NEXT CLASS** | **FRAMES**    **NO FRAMES**     **All Classes** |  |
| SUMMARY: NESTED \| FIELD \| CONSTR \| METHOD | DETAIL: FIELD \| CONSTR \| METHOD |  |


---


## org.proteinshader.structure Class Category

```
java.lang.Object
  org.proteinshader.structure.Category
```

---

``` public class Category extends Object ```

A Category object serves as a container for the Records in a PDB
structure entry.

---

**Constructor Summary**

`Category(String name)`
  
          Constructs a Category with the name specified as an argument.


| **Method Summary** | |
| --- | --- |
| `Record` | `addNewRecord(String recordName)`             Creates a Record, adds it to a hash, and returns the new Record. |
| `String` | `getName()`             Returns the name of the Category. |
| `Record` | `getRecord(String recordName)`             Retrieves a Record by its name. |
| `Iterator<Record>` | `iteratorRecords()`             Returns an Iterator for the Records held by this Category object. |
| `int` | `numberOfRecords()`             Returns the number of Records held by this Category. |
| `void` | `removeRecord(String recordName)`             Removes a Record. |

**Methods inherited from class java.lang.Object**

`clone, equals, finalize, getClass, hashCode, notify, notifyAll, toString, wait, wait, wait`

**Constructor Detail**

### Category

```
public Category(String name)
         throws InvalidIDException
```

:   Constructs a Category with the name specified as an argument.
    An InvalidIDException will be thrown if name does not have at
    least one character.

    **Parameters:**: `name` - the name of the Category. **Throws:**: `InvalidIDException` - if name is null or an empty String.


**Method Detail**

### getName

```
public String getName()
```

:   Returns the name of the Category.
    The Category name cannot be null or an empty String because is
    checked by the constructor and is read-only.

    :   **Returns:**: The Category name.

---


### addNewRecord

```
public Record addNewRecord(String recordName)
                    throws InvalidIDException
```

:   Creates a Record, adds it to a hash, and returns the new Record.
    The Record will be marked with the name of the Category object
    that owns it.

    :   **Parameters:**: `recordName` - the name of the Record. **Returns:**: The new Record. **Throws:**: `InvalidIDException` - if recordName is null or an empty String.

---


### getRecord

```
public Record getRecord(String recordName)
```

:   Retrieves a Record by its name.
    Returns null if the requested Record does not exist.

    :   **Parameters:**: `recordName` - the name of the Record. **Returns:**: The requested Record.

---


### removeRecord

```
public void removeRecord(String recordName)
```

:   Removes a Record.
    No action is taken if the Record does not exist.

    :   **Parameters:**: `recordName` - the name of the Record.

---


### iteratorRecords

```
public Iterator<Record> iteratorRecords()
```

:   Returns an Iterator for the Records held by this Category object.
    The iteration order will be the same as the order in which the
    Records were added to the Category. If a Record is overridden
    with another Record of the same name, the replacement would not
    change the iteration order in any way.

    :   **Returns:**: An Iterator for the Records.

---


### numberOfRecords

```
public int numberOfRecords()
```

:   Returns the number of Records held by this Category.

    :   **Returns:**: The total number of Records.


---


|  |  |  |
| --- | --- | --- |
| **Overview** \| **Package** \| **Class** \| **Use** \| **Tree** \| **Deprecated** \| **Index** \| **Help** | |  |
| **PREV CLASS**   **NEXT CLASS** | **FRAMES**    **NO FRAMES**     **All Classes** |  |
| SUMMARY: NESTED \| FIELD \| CONSTR \| METHOD | DETAIL: FIELD \| CONSTR \| METHOD |  |


---

# *Copyright © 2007-2008*
